# Comparison of Latent Tuberculosis Infections among General versus Tuberculosis Health Care Workers in Myanmar

**DOI:** 10.3390/tropicalmed5030116

**Published:** 2020-07-14

**Authors:** Moe Hnin Phyu, Hutcha Sriplung, Myo Su Kyi, Cho Cho San, Virasakdi Chongsuvivatwong

**Affiliations:** 1 National Tuberculosis Programme, Ministry of Health and Sports, Nay Pyi Taw Union Territory 15011, Myanmar; dr.moehninphyu.mhp@gmail.com (M.H.P.); dr.ccs.ntp@gmail.com (C.C.S.); 2 Epidemiology Unit, Faculty of Medicine, Prince of Songkla University, Hat Yai, Songkhla 90110, Thailand; 3 Regional Public Health Department, Nay Pyi Taw Union Territory 15011, Myanmar; drmyosukyi@gmail.com

**Keywords:** latent TB infection, health care worker, tuberculosis

## Abstract

Health care workers (HCWs) in high tuberculosis (TB) prevalence countries have to care for many cases, thus increasing their risk of infection. The objective of the study was to compare the prevalence of latent TB infection (LTBI) between general HCWs and TB HCWs, and also to explore the associated factors. A cross-sectional study was conducted in Nay Pyi Taw, Myanmar from September 2019 to January 2020. Staff working at two general hospitals were recruited. Those allocated for TB care were classified as TB HCWs, while the remaining were classified as general HCWs. Participants were interviewed using a structured questionnaire, and screened for LTBI using a tuberculin skin test (TST). Individuals who had an induration of 10 mm or more with normal chest radiograph were regarded as having LTBI. The prevalence of LTBI among general HCWs was 2.04 times higher than that of TB HCWs (31.2% vs. 15.3%, *p* < 0.001). The associated factors for LTBI included low education level, duration of work experience ≥ 10 years, a low knowledge of regular TB screening, and teaching cough etiquette to TB patients. The higher prevalence of LTBI in the general HCWs in this study was due to confounding by education and experience. After adjustment for these, we have no evidence to support that either group of HCWs had higher LTBI risk.

## 1. Introduction

Tuberculosis is an airborne infectious disease caused by *Mycobacterium tuberculosis* [1]. An individual can have an asymptomatic latent TB infection (LTBI) after inhalation of infectious droplets [2,3]. About 5–10% of LTBI individuals will eventually develop an active TB disease [4,5]. Individuals with LTBI will not show any clinical, bacteriological or radiological features. The tuberculin skin test (TST) is one of the diagnostic tests for latent TB [6].

TB has been a top-priority health problem of Myanmar. Bacillus Calmette–Guérin (BCG) vaccination has become part of the Expanded Program of Immunization in Myanmar since 1978. BCG vaccine is administered to all newborns at birth, and the current coverage is estimated to be 86% [7]. Nay Pyi Taw is one of the high TB burden areas, with a notification rate of 269 per 100,000 people in 2017.

Health care workers (HCWs) in high TB prevalence countries have to care for many cases, thus increasing their risk of infection irrespective of their place of employment [8,9,10]. The prevalence of LTBI among HCWs ranged from 10% to 62%, depending on the specific TB burden of the country and the workplace of the HCWs [4,11,12,13]. The prevalence among HCWs at a general hospital in India was 36.8% [11], while the prevalence among TB HCWs was as great as 51.4% in China [12].

According to studies from India and China, TB HCWs have a higher LTBI prevalence than general HCWs. Therefore, the study aimed to compare the prevalence of LTBI between general HCWs and TB HCWs, and determine factors associated with LTBI among all HCWs.

## 2. Materials and Methods

### 2.1. Study Design and Setting

A cross-sectional study was conducted in Nay Pyi Taw, Myanmar, from September 2019 to January 2020 at two general hospitals. TB control activities are conducted at public health TB clinics. There are no specialized TB hospitals in the study area, but all hospitals provide outpatient care for TB patients. Staff working at the general hospital were approached and recruited to the study. Those who were involved in caring for TB patients were classified as TB HCWs while the remaining were classified as general HCWs.

### 2.2. Ethics Approval

The Institutional Ethics Committee of the Faculty of Medicine, Prince of Songkla University, Hat Yai, Thailand (62-263-18-1) and the Institutional Review Board (1), Ministry of Health and Sports, Myanmar (2019-010) gave ethical approval to conduct the study. Informed written consent was obtained from all participants.

### 2.3. Study Participants and Methods

TB HCWs included team leaders, nurses, TB coordinators, TB clinic staff and basic health staff. The last categories are working contact tracing, directly observed treatment of TB patient and multidrug-resistant (MDR) TB patients. General HCWs included doctors, nurses, pharmacists, technicians, administrative staff and ward staff who were not directly responsible for TB care. The two independent proportion formula was used to calculate the sample size. The estimated difference in risk of LTBI between TB and general HCWs was set at 15% (51% [12] vs. 36% [11]). Given a type-I error of 5%, a power of 80%, and a ratio of 1, at least 184 TB HCWs and 184 general HCWs were required. Invitation letters enclosed with the study information sheets were sent to all eligible TB and general HCWs in the study townships and those agreeing to join were recruited into the study. However, HCWs who were absent or on leave during the study period were excluded.

### 2.4. Questionnaire Preparation

A questionnaire containing questions related to TB infection control (TBIC), knowledge and practice were prepared in line with the World Health Organization (WHO) TB infection control measures, and the related literature [14,15,16]. Modification into the local context was done with help from TB technical experts. The questionnaire was prepared in English, translated into the Myanmar language, and then translated back into English by another independent translator. Pre-testing was done among HCWs who were not included in the actual study. Internal consistency was measured using the Kuder–Richardson test and its value was 0.72.

### 2.5. Data Collection

Interviewer training was given to three research assistants by the principal investigator. For TST, a senior consultant microbiologist provided training to four nurses and two TB clinic doctors. The potential participants were informed about the study, including the need for TST. After providing informed consent, the participants were interviewed using the structured questionnaire, containing demographic characteristics, previous exposure to TB, and knowledge and practice on TB infection control measures. After the interview, 0.1 mL (2 IU) of tuberculin purified protein derivative (Thai Red Cross Society Tuberculin PPD, Product code FT006, Lot No.TP00119) was administered using the Mantoux method. Skin reaction was measured 48–72 h later by TB clinic doctors, and individuals with ≥10 mm induration were further investigated with chest radiography to rule out active TB.

### 2.6. Variables and Measurements

The dependent variable was whether the HCW had a positive TST result. The primary independent variable was the type of HCW (general versus TB), and the potential confounders were age in years, duration of service in years, history of previous exposure to TB patients, and knowledge and practice of TB infection control measures. Exposure to TB cases was assessed by asking the questions regarding number of presumptive TB encounters per day, number of TB patients encountered per day and number of MDR TB patients encountered per day. HCWs who had TST results with an induration ≥ 10 mm and no features consistent with TB on the chest X-ray were classified as having LTBI.

### 2.7. Statistical Analysis

Double data entry was done using EpiData. All statistical analyses were performed using R (version 3.6.2). Categorical variables were summarized as percentages and continuous variables were presented as the median and interquartile range (IQR). The Mann–Whitney test was used to compare the difference between the continuous variables of general and TB HCWs. Fisher’s exact test and the Chi square test were used to determine the strength of association between the categorical variables and the outcome. To determine the associated factors of LTBI among HCWs, variables with a *p*-value < 0.20 in the univariate analysis were included in a multivariable logistic regression model. Statistical significance was 5%.

## 3. Results

**Figure 1:**
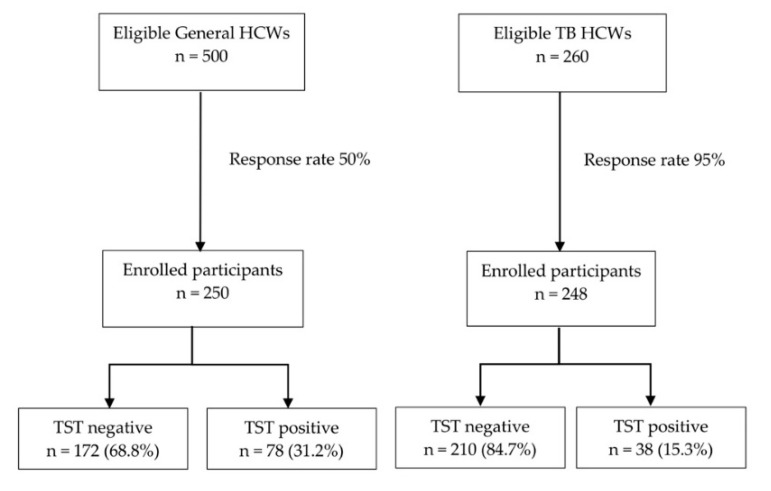
Flow of the study showing LTBI of HCWs.

Figure 1 summarizes the flow of the study. In total, 500 general and 260 TB HCWs were eligible for the study, of which 250 general and 248 TB HCWs agreed to participate in the study, giving response rates of 50% and 95%, respectively. We identified 116 TST positive individuals; 78 among general and 38 among TB HCWs.

### 3.1. Background Characteristics of HCWs

The background characteristics of the general and TB HCWs are compared in Table 1. General HCWs were older, less educated, had a longer service duration, a wider variety of professions, and were less exposed to TB compared to TB HCWs.

### 3.2. Prevalence of LTBI and Associated Factors

Table 1 also shows the factors associated with LTBI via univariate analysis. The prevalence of LTBI among general HCWs was significantly higher than it was among TB HCWs (31.2% vs. 15.3%, *p* < 0.001). Other factors significantly associated with LTBI were age, education, duration of work experience, type of profession and level of exposure to presumptive TB.

Table 2 shows the association between TB infection control knowledge and LTBI status. The percentage of HCWs who were diagnosed with LTBI was significantly lower for those who knew the following four facts: (1) minimizing contact time with TB patients is necessary for TB control, (2) screening HCWs for TB is one of the TB infection control measures, (3) doors and windows of a room should be opened whenever a TB case is present, and (4) fans can be used to reduce TB transmission.

Table 3 presents univariate analysis of the effect of HCWs’ practice on TB infection control and LTBI status. The percentage of HCWs who were diagnosed with LTBI was significantly lower for those who reported practicing the following two behaviors: (1) prioritizes coughing patients, and (2) opens the windows whenever a TB case is in the room.

Table 4 shows the results of the final multivariable logistic regression model predicting LTBI among the HCWs. The type of HCWs was not significant after adjusting for other variables. The associated factors for LTBI among HCWs were not being a graduate (AOR: 1.78, 95% CI: 1.02–3.1), service years of 10 years or more (AOR: 3.23, 95% CI: 1.93–5.38), having poor knowledge of regular TB screening (AOR: 0.28, 95% CI: 0.15–0.54) and teaching cough etiquette to TB patients (AOR: 7.38, 95% CI: 2.61–20.86).

## 4. Discussion

This study found that the prevalence of LTBI among general HCWs was double that among TB HCWs. The risk factors of LTBI included having a less than tertiary level of education, having 10 or more years of service, having poor knowledge of regular TB screening, and teaching cough etiquette to TB patients.

The prevalence of LTBI among general HCWs is similar to the prevalence among the general population in the Southeast Asia region [17]. The study in Georgian HCWs found that the prevalence of LTBI was higher among HCWs in TB facilities than non-TB facilities (69% vs. 54%) [18]. However, South Korean studies showed that there was no difference in LTBI prevalence between TB related HCWs and general HCWs [9,19]. Similarly, our study found that the type of HCWs was no longer significant after adjusting the other confounding variables. This finding may diminish the concern of HCWs who are reluctant to work in TB-specialized health care settings [20]. The study reflects that infection control is essential in all health care settings, whether it is TB specialty or not.

Having a less than tertiary education level was an independent factor for LTBI (OR = 1.78). Furthermore, HCWs with a low background education might have less understanding of infection control measures, and therefore a specific policy of using tailored courses and printed materials may be required to promote knowledge and practice among this subset of HCWs [16].

Increases in duration of service may increase the frequency of contact with the patients, and this was associated with LTBI in this study (OR = 3.23). Previous studies also found that the frequency of contact with TB patients increased the risk of TB infection [18,21]. We observed that HCWs who were exposed to TB cases were less likely to be infected with TB, although it was not significant in the adjusted analysis. The reason may be that individuals who can recall exposure tend to be the ones who pay more attention to not spreading TB.

Regarding TB infection control, HCWs who do not know that regular TB screening is an infection control measure were more likely to have LTBI. This finding supports the establishment of a policy of annual screening of TB for all HCWs in Myanmar. Standard TB infection control measures include educating TB patients on cough etiquette in order to prevent the spread of infectious droplets in health facilities [15]. However, HCWs whose main job involved providing education on cough etiquette were more likely to have LTBI (OR = 7.38). This may be due to HCWs not having adequate self-protection during their education sessions. However, further studies are needed to confirm this theory.

Previous studies found that household contacts had a high risk of latent TB infection [22]. This was not the case in our study, mainly because of the low percentage of exposure in both groups. BCG vaccination has been known to cause a false positive TST result, and to have a limited protective effect on TB [23,24]. Our data, through univariate analysis, suggest that those vaccinated were less likely to have a positive TST result. This was probably due to socio-economic confounders. However, multivariate analysis diminishes such difference.

Finally, for HCWs who had positive TST, WHO recommended that LTBI may not require treatment if TB prevalence in the country is high. Since this is the situation in Myanmar, we did not offer the treatment, but advised them to get yearly follow-ups, or to do so earlier if there is any suspicion.

There were some limitations in our study. This study was subject to volunteer selection bias at the time of recruiting participants. Non-response bias is also an issue as 50% of the eligible general HCWs refused to participate in the study. General HCWs were mostly on duty and unavailable to participate in the study, and those who were available may overestimate or underestimate their LTBI status. Moreover, we did not perform any HIV testing to confirm the participants HIV status. This limitation indicates a caution about false negative TST results.

## 5. Conclusions

The higher prevalence of LTBI in the general HCWs in this study was due to confounding by education and experience. After adjustment for these, we have no evidence to support that either group of HCWs had higher LTBI risk. It is important to evaluate the TB infection control measures in all of the health care facilities, and establish the TB surveillance system among HCWs.

## Figures and Tables

**Table 1:** Background characteristics of HCWs.

Variable	Total N = 498, n (%)	General HCW N = 250, n (%)	TB HCW N = 248, n (%)	*p*-Value	TST Negative N = 382, n (%)	TST Positive N = 116, n (%)	*p*-Value
HCW Type							<0.001
TB	248 (49.8)	-	-		210 (84.7)	38 (15.3)	
General	250 (50.2)	-	-		172 (68.8)	78 (31.2)	
Age, years, Median (IQR)	26 (23, 35)	31 (25, 42)	25 (23, 28)	<0.001	26 (23, 32)	33.5 (25, 43)	<0.001
Age group, years				<0.001			<0.001
20–29	327 (65.7)	124 (49.6)	203 (81.9)		277 (72.5)	50 (43.1)	
30–39	92 (18.5)	60 (24.0)	32 (12.9)		62 (16.2)	30 (25.9)	
40–49	64 (12.9)	55 (22.0)	9 (3.6)		34 (8.9)	30 (25.9)	
50+	15 (3)	11 (4.4)	4 (1.6)		9 (2.4)	6 (5.2)	
Gender				0.694			0.596
Female	393 (78.9)	195 (78.0)	198 (79.8)		304 (79.6)	89 (76.7)	
Male	105 (21.1)	55 (22.0)	50 (20.2)		78 (20.4)	27 (23.3)	
Graduated				<0.001			<0.001
Yes	390 (78.3)	154 (61.6)	236 (95.2)		316 (82.7)	74 (63.8)	
No	108 (21.7)	96 (38.4)	12 (4.8)		66 (17.3)	42 (36.2)	
Profession				<0.001			<0.001
Administrative staff	30 (6.0)	30 (12.0)	0 (0)		20 (5.2)	10 (8.6)	
Basic Health staff	230 (46.2)	4 (1.6)	226 (91.1)		194 (50.8)	36 (31.0)	
Doctor/Nurse/Lab staff	158 (31.7)	136 (54.4)	22 (8.9)		120 (31.4)	38 (32.8)	
Pharmacist	8 (1.6)	8 (3.2)	0 (0)		2 (0.5)	6 (5.2)	
Ward staff	72 (14.5)	72 (28.8)	0 (0)		46 (12.0)	26 (22.4)	
Total service years, Median (IQR)	4 (3, 10)	6 (3,15)	4 (3,6)	<0.001	4 (3, 6)	7.5 (4, 16)	<0.001
Total service years				<0.001			<0.001
<10	390 (78.3)	166 (66.4)	224 (90.3)		322 (84.3)	68 (58.6)	
10+	108 (21.7)	84 (33.6)	24 (9.7)		60 (15.7)	48 (41.4)	
Exposure to presumptive TB cases				<0.001			<0.001
No	216 (43.4)	180 (72.0)	36 (14.5)		148 (38.7)	68 (58.6)	
Yes	282 (56.6)	70 (28.0)	212 (85.5)		234 (61.3)	48 (41.4)	
Exposure to TB patients				<0.001			0.006
No	234 (47)	178 (71.2)	56 (22.6)		166 (43.5)	68 (58.6)	
Yes	264 (53)	72 (28.8)	192 (77.4)		216 (56.5)	48 (41.4)	
Exposure to MDR TB patients				0.876			0.961
No	392 (78.7)	198 (79.2)	194 (78.2)		300 (78.5)	92 (79.3)	
Yes	106 (21.3)	52 (20.8)	54 (21.8)		82 (21.5)	24 (20.7)	
Previous BCG vaccination				<0.001			0.005
No	208 (41.8)	148 (59.2)	60 (24.2)		146 (38.2)	62 (53.4)	
Yes	290 (58.2)	102 (40.8)	188 (75.8)		236 (61.8)	54 (46.6)	
Household TB contact				1			0.336
No	450 (90.4)	226 (90.4)	224 (90.3)		342 (89.5)	108 (93.1)	
Yes	48 (9.6)	24 (9.6)	24 (9.7)		40 (10.5)	8 (6.9)	
Previous history of TB				0.751			0.99
No	488 (98)	244 (97.6)	244 (98.4)		374 (97.9)	114 (98.3)	
Yes	10 (2)	6 (2.4)	4 (1.6)		8 (2.1)	2 (1.7)	

HCW: health care worker; IQR: interquartile range; MDR TB: multi-drugs resistant TB; TST: tuberculin skin test; BCG: Bacillus Calmette–Guérin.

**Table 2:** Univariate analysis of TB infection control knowledge and LTBI among HCWs.

Knowledge Item	TST Negative N = 382, n (%)	TST Positive N = 116, n (%)	*p*-Value
An infection control committee is necessary for TB control			1
No	40 (10.5)	12 (10.3)	
Yes	342 (89.5)	104 (89.7)	
Minimizing contact time with TB patients is necessary for TB control			<0.001
No	24 (6.3)	20 (17.2)	
Yes	358 (93.7)	96 (82.8)	
Presumptive or confirmed TB cases should be separated			0.82
No	22 (5.8)	8 (6.9)	
Yes	360 (94.2)	108 (93.1)	
TB patients should be educated on cough etiquette			0.99
No	14 (3.7)	4 (3.4)	
Yes	368 (96.3)	112 (96.6)	
Presumptive or confirmed TB cases who are coughing should be given priority			0.807
No	38 (9.9)	10 (8.6)	
Yes	344 (90.1)	106 (91.4)	
TB screening of HCWs is one of the TB infection control measures			<0.001
No	44 (11.5)	34 (29.3)	
Yes	338 (88.5)	82 (70.7)	
The doors and windows of a room should be open whenever a TB case is present			<0.001
No	80 (20.9)	46 (39.7)	
Yes	302 (79.1)	70 (60.3)	
Fans can be used to reduce TB transmission			<0.001
No	110 (28.8)	56 (48.3)	
Yes	272 (71.2)	60 (51.7)	
Surgical masks cannot protect HCWs from getting infected with TB			0.479
No	142 (37.2)	48 (41.4)	
Yes	240 (62.8)	68 (58.6)	
Respirators can protect HCWs from getting infected with TB			0.864
No	64 (16.8)	18 (15.5)	
Yes	318 (83.2)	98 (84.5)	

TST: tuberculin skin test; HCW: health care worker.

**Table 3:** Univariate analysis of TB infection control practices associated with LTBI among HCWs.

Behavior Item	TST Negative N = 382, n (%)	TST Positive N = 116, n (%)	*p*-Value
Prioritizes coughing patients			0.02
No	100 (26.2)	44 (37.9)	
Yes	282 (73.8)	72 (62.1)	
Educates TB patients about correct cough etiquette			0.123
No	40 (10.5)	6 (5.2)	
Yes	342 (89.5)	110 (94.8)	
Tests for TB in case you have cough			0.337
No	118 (30.9)	42 (36.2)	
Yes	264 (69.1)	74 (63.8)	
Opens the windows whenever a TB case is in the room			0.02
No	112 (29.3)	48 (41.4)	
Yes	270 (70.7)	68 (58.6)	
Turns on the fan while treating TB cases			0.342
No	196 (51.3)	66 (56.9)	
Yes	186 (48.7)	50 (43.1)	
Uses surgical mask whenever treating TB patients			0.716
No	52 (13.6)	18 (15.5)	
Yes	330 (86.4)	98 (84.5)	
Uses N95 whenever treating TB patients			0.626
No	252 (66)	80 (69)	
Yes	130 (34)	36 (31)	

TST: tuberculin skin test.

**Table 4:** Multivariate analysis of factors associated with LTBI among HCWs.

Variable	Crude OR (95% CI)	Adj. OR (95% CI)	*p*-Value
Type of HCW: General HCW vs. TB HCW	2.51 (1.62, 3.88)	1.37 (0.8, 2.33)	0.253
Graduated: No vs. Yes	2.72 (1.71, 4.31)	1.78 (1.02, 3.1)	0.043
Total service years: 10+ vs. <10	3.79 (2.39, 6.01)	3.23 (1.93, 5.38)	<0.001
Knowing that TB screening of HCWs is a TB infection control measure: Yes vs. No	0.31 (0.19, 0.52)	0.28 (0.15, 0.54)	<0.001
Educating cough etiquette to TB patients: Yes vs. No	2.14 (0.89, 5.19)	7.38 (2.61, 20.86)	<0.001

OR: odds ratio; CI: confidence interval; HCWs: health care workers.
